# Supplementary material for: Altered Cell Surface N-Glycosylation of Resting and Activated T Cells in Systemic Lupus Erythematosus
Source: Int J Mol Sci. 2019 Sep 10;20(18):4455. doi: 10.3390/ijms20184455 (PMC6770513; doi:10.3390/ijms20184455)
Supplement: Supplementary file 1 [file ijms-20-04455-s001.pdf]

Supplementary Data

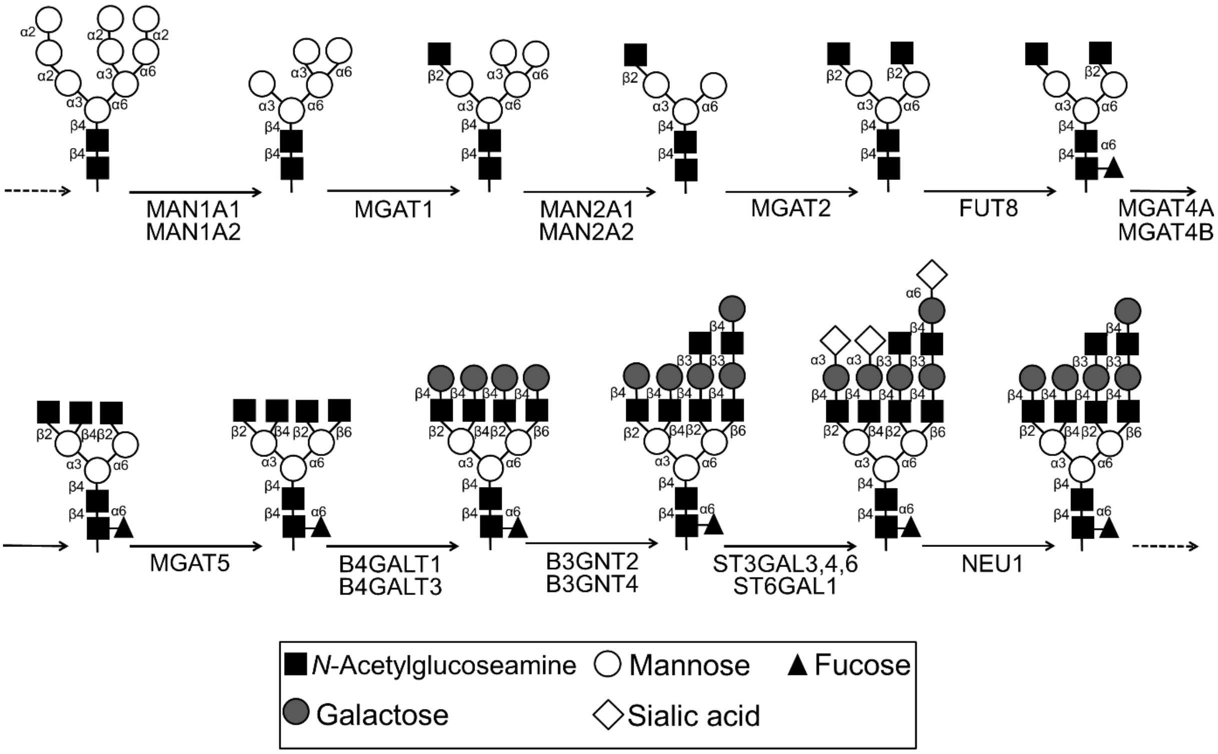

Figure S1. Schema of the protein N-linked glycosylation pathway in mammals**.** Human gene symbols encoding glycan modifying enzymes are shown below the arrows. For the explanation of the abbreviations of enzyme names, please refer to Table 2.

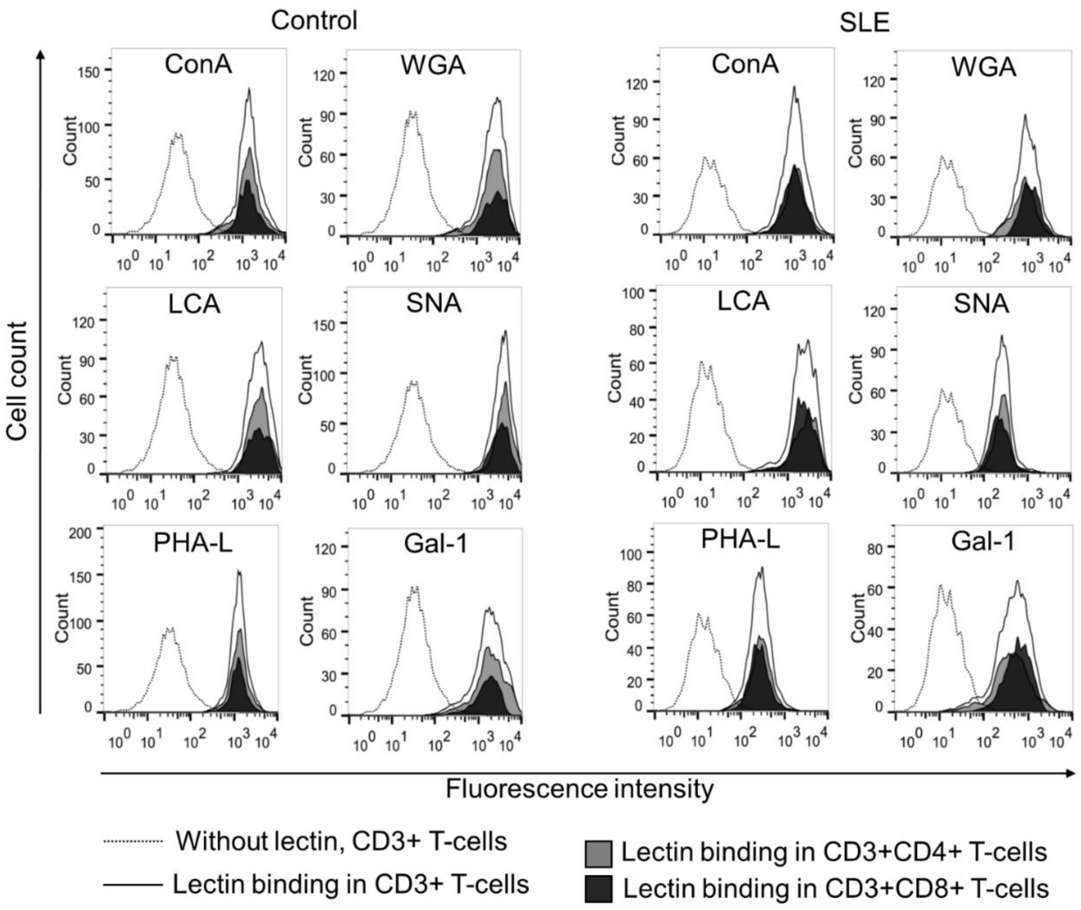

Figure S2. Lectin binding in CD4+ or CD4- (i.e., CD8+) T cells from healthy individuals and SLE patients. Peripheral blood T cells were obtained from healthy controls and SLE patients. The cells were activated with 1 μg/mL phytohaemagglutinin L (PHA-L) for 72 hours. Activated cells were stained with viability dye, fixed, then labeled with anti-CD3-PE-Cy5 and anti-CD4-PE antibody(BioLegend), followed by FITC-conjugated lectins, and were evaluated by flow cytometry. Representative histograms of three independent experiments show the lectin binding of all CD3+ T cells (black lined white area), the CD3+CD4+ T cell subpopulation (grey shadowed area), and the CD3+CD4- (considered as CD8+) T cell subpopulation (black shadowed area).

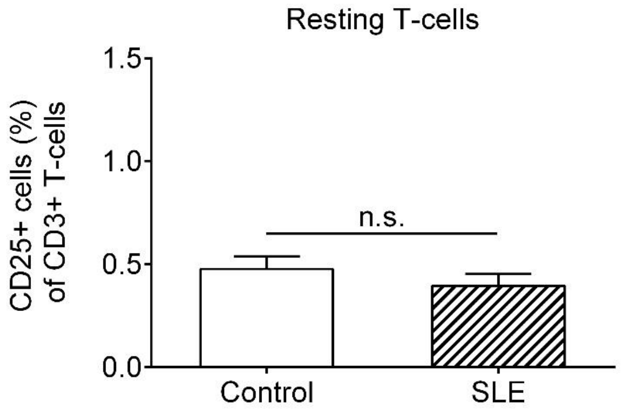

Figure S3. Ratio of CD25-positive cells among resting T cells**.** Peripheral blood mononuclear cells were obtained from healthy controls and SLE patients. PBMCs were stained with viability dye, fixed, then labeled with anti-CD3-PE-Cy5 antibody and anti-CD25-PE antibody (BioLegend), and evaluated by flow cytometry as described in the lectin binding assay section of the Materials and Methods. The percentage of CD25-positive cells within the CD3-positive living T cell population is shown. Statistical analysis was performed using an unpaired *t*-test, n.s. = not significant.
